# Can depression lead to chronic constipation, or does chronic constipation worsen depression? NHANES 2005–2010 and bidirectional mendelian randomization analyses

**DOI:** 10.1186/s12876-024-03454-x

**Published:** 2024-10-10

**Authors:** ZhiGuo He, QianLe Yu, Bin He, JieFeng Liu, WenBin Gao, Xiong Chen

**Affiliations:** Department of General Surgery, Affiliated Changsha Hospital of Hunan Normal University, No. 70, Lushan Road, Changsha, 410000 PR China

**Keywords:** Depression, Chronic constipation, NHANES, Mendelian randomization, Reverse causation

## Abstract

**Background:**

Depression and chronic constipation often co-occur, but the reciprocal influence between the two remains unclear. The purpose of this study is to explore the potential association between depression and chronic constipation.

**Methods:**

This study initially utilized data from National Health and Nutrition Examination Survey (NHANES) 2005–2010 to explore the correlation between depression scores and chronic constipation, assessing the non-linear relationship between the two. Subsequently, we conducted a two-sample Mendelian randomization (MR) analysis to evaluate the causal relationship between depression and major depression with chronic constipation. The Inverse Variance Weighting (IVW) method served as the primary reference, supplemented by sensitivity tests. Finally, a reverse MR analysis was performed to assess the presence of any reverse causation. The STROBE-MR checklist for the reporting of MR studies was used in this study.

**Results:**

In the NHANES analysis, survey-weighted logistic regression revealed a significantly positive correlation between depression scores and chronic constipation (OR = 1.04, 95% CI = 1.02–1.07, *p* = 0.002), even after adjusting for the included covariates. The nonlinear analysis using Restricted Cubic Splines (RCS) enhanced the robustness of the association (P-non-liner = 0.01). The MR analysis also confirmed the causal relationship between depression (OR = 11.43, 95% CI = 1.85–70.67, *p* = 0.008) and major depression (OR = 1.12, 95% CI = 1.03–1.22, *p* = 0.007) with chronic constipation, passing rigorous sensitivity tests. No evidence of reverse causation was observed in the reverse MR analysis (*P* > 0.05).

**Conclusions:**

Depression is positively correlated with the risk of chronic constipation. Therefore, enhancing attention to chronic constipation in patients with depression may be effective in clinical practice.

**Supplementary Information:**

The online version contains supplementary material available at 10.1186/s12876-024-03454-x.

## Introduction

Depression is a common chronic mental illness associated with an increased risk of suicide [1]. The number of people suffering from depression worldwide is as high as 300 million, making it the most disabling disease, according to the World Health Organization [2]. This should be of great concern, especially regarding major depression in adolescents [3]. Early clinical intervention can effectively prevent premature deaths among young people [4]. Additionally, adults are also seriously affected by depression. Research has shown that depression significantly increases the overall mortality rate and the mortality rate from cardiovascular diseases among Chinese people [5]. Given the serious threat of depression, early intervention and prevention are particularly important.

Chronic constipation is a common gastrointestinal disorder that affects individuals of all ages [6]. Many medications are available for the treatment of chronic constipation, and conservative measures are reasonably effective, with only a small percentage of patients seeking medical assistance [7, 8]. However, many chronic constipation sufferers do not respond to drug therapy [9]. If left untreated, chronic constipation can progress to serious gastrointestinal conditions such as fecal impaction and intestinal perforation [10]. Research has found that chronic chronic constipation exacerbates urological and reproductive system disorders and is highly correlated with cardiovascular and cerebrovascular diseases, posing a significant threat to human health [11, 12]. The causes of chronic constipation are complex and multifaceted, and it is generally believed to be influenced by factors such as diet, stress, medication, and neurological factors [13]. Many scholars have suggested that psychological distress and mental factors may be associated with the occurrence of chronic constipation [14, 15].

Interestingly, depression often co-occurs with chronic constipation, and individuals with depression seem to be more susceptible to chronic constipation [16]. However, the underlying mechanisms of this bidirectional causation remain unclear. Furthermore, while many studies have proposed a correlation between depression and chronic constipation, the influence of the severity of depression on chronic constipation, or whether persistent chronic constipation exacerbates depression, is a seldom-addressed question. Major depression is more prone to generating suicidal thoughts, especially among adolescents [17, 18]. Adolescents with chronic constipation often exhibit noticeable emotional issues, but there is no evidence suggesting a connection between chronic constipation and the severity of depression or suicidal tendencies [19, 20]. Based on this, we incorporated both depression and major depression into the study, aiming to elucidate the potential relationship between them.

NHANES comprises questionnaire surveys and clinical information targeting the U.S. population, providing a nationally representative and reliable large-scale dataset widely utilized in epidemiological research [21]. MR is an emerging causal analysis method. Based on the principle that genetic variations determine phenotypes, this method employs single nucleotide polymorphisms (SNPs) highly correlated with the exposure as instrumental variables (IVs), conducting unidirectional causal analysis from exposure to outcome [22]. Due to its independence from environmental influences, it effectively addresses confounding factors and interference from reverse causation [23, 24]. MR must satisfy three conditions: (1) IVs are strongly correlated with the exposure, (2) IVs are uncorrelated with confounding factors, (3) IVs affect the outcome only through the exposure [25].

Our aim is to investigate the bidirectional causal relationship between depression and chronic constipation, including the influence of depression severity. We combine a large-scale analysis of NHANES data (2005–2010) with bidirectional two-sample MR analysis to explore the potential associations between depression and chronic constipation from both a cross-sectional and causal perspective. This study may offer new insights into the prevention of depression or chronic constipation and provide clinicians with a fresh perspective on management.

## Methods

### Participants from the NHANES database

Our study included a total of 11,760 participants from three NHANES cycles (2005–2010). All of them provided informed consent, so there were no ethical issues involved. The exclusion criteria were as follows: (1) Missing data on common stool types and depression scores; (2) Missing data on covariates such as gender, age, race, marital status, education, poverty income ratio (PIR), Body Mass Index (BMI), physical activity, and dietary intake; (3) Participants who were pregnant; (4) Participants with a history of colorectal cancer; (5) Common stool type recorded as unknown or diarrhea; (6) Unknown milk consumption and physical activity status. For specific details, please refer to Supplementary Figure S1.

### Depression scores and common stool types

Depression scores were assessed using the Patient Health Questionnaire (PHQ), which has been proven to be a reliable method for diagnosing depression [26]. Based on questions about 9 symptoms and signs of depression over the past 2 weeks (such as mood, sleep, mental state, appetite, and suicidal tendencies), each item is scored from 0 (not at all) to 3 (nearly every day), resulting in a total score ranging from 0 to 27. We defined a total depression score of < 10 as no depression, >=10 as depression, and > = 15 as major depression.

The Bristol Stool Form Scale (BSFS) was used to define chronic constipation [27]. Participants identified their common stool type by viewing cards with different stool types. Type 1 (separate hard lumps, like nuts) and Type 2 (sausage-shaped but lumpy) were defined as chronic constipation. Types 3 (sausage-like but with cracks on the surface), 4 (sausage or snake-shaped, smooth and soft), and 5 (soft blobs with clear-cut edges) were defined as normal stool. Type 6 (fluffy pieces with ragged edges, a mushy stool) and Type 7 (watery, no solid pieces) are defined as chronic diarrhea. To avoid selection bias, we conducted independent participant exclusion for chronic diarrhea and used adjusted weighted logistic regression to verify whether depression increases the risk of chronic diarrhea. The results indicate that depression is also a risk factor for chronic diarrhea (Supplementary Table S1). Therefore, to obtain a more accurate correlation between depression and chronic constipation, we excluded participants defined as having chronic diarrhea or unknown bowel conditions.

### Covariates

As shown in Table 1, demographic variables include age, gender, race (Mexican American, Non-Hispanic White, Non-Hispanic Black, Other Hispanic, and Other/Multiracial), PIR, education attainment (Less Than 9th Grade, 9-11th Grade, High School Grad/GED, Some College or AA degree, and College Graduate or above), and marital status. Other variables include BMI (Underweight, Normal, Overweight, and Obese), smoking status (Never smoker, Former smoker, and Current smoker), alcohol consumption (1–5 drinks/month, 5–10 drinks/month, 10 + drinks/month, and Non-drinker), physical activity (non-vigorous activities and vigorous activities), milk consumption (never, rarely, sometimes, and often), and the average of two consecutive days of dietary intake (total calories, protein, Carbohydrate, Sugars, Fiber, fat, cholesterol, Moisture, and Caffeine).

**Table 1:** Basic information of the included population, classified by chronic constipation and non-chronic constipation

Characteristic	Chronic constipation		*P* Value^2^
	No, *N* = 10,808 (93%)^1^	Yes, *N* = 952 (7.3%)^1^	
**Age (years)**			0.2
*20–39 years*	3,477 (35%)	336 (37%)	
*40–59 years*	3,484 (38%)	305 (36%)	
*60–79 years*	2,816 (19%)	211 (17%)	
*80 + years*	1,031 (7.7%)	100 (9.4%)	
**Gender**			**< 0.001**
*female*	5,114 (49%)	639 (71%)	
*male*	5,694 (51%)	313 (29%)	
**Race**			**< 0.001**
*Mexican American*	1,856 (7.4%)	172 (10%)	
*Other Hispanic*	815 (3.8%)	98 (4.8%)	
*Non-Hispanic White*	5,589 (73%)	426 (65%)	
*Non-Hispanic Black*	2,112 (10%)	224 (15%)	
*Other/multiracial*	436 (5.1%)	32 (4.8%)	
**PIR**			**< 0.001**
*< 1*	1,982 (12%)	221 (17%)	
*>=1*	8,826 (88%)	731 (83%)	
**Education.attainment**			**< 0.001**
*Less Than 9th Grade*	1,073 (4.7%)	132 (8.4%)	
*9-11th Grade*	1,686 (12%)	181 (16%)	
*High School Grad/GED*	2,571 (24%)	260 (29%)	
*Some College or AA degree*	3,145 (32%)	226 (26%)	
*College Graduate or above*	2,327 (28%)	151 (21%)	
**Marital.Status**			**0.007**
*Living alone*	4,130 (36%)	418 (42%)	
*Married or living with partner*	6,673 (64%)	534 (58%)	
**BMI.group**			**< 0.001**
*Underweight(< 18.5)*	157 (1.6%)	24 (2.2%)	
*Normal(18.5 to < 25)*	2,951 (30%)	316 (37%)	
*Overweight(25 to < 30)*	3,723 (34%)	313 (33%)	
*Obese(30 or greater)*	3,977 (35%)	299 (27%)	
**Smoking status**			**0.012**
*Never smoker*	5,604 (52%)	563 (58%)	
*Former smoker*	2,782 (25%)	195 (20%)	
*Current smoker*	2,419 (23%)	194 (22%)	
**Drinking status**			**< 0.001**
*1–5 drinks/month*	5,294 (49%)	451 (49%)	
*5–10 drinks/month*	938 (10%)	54 (6.7%)	
*10 + drinks/month*	1,715 (19%)	90 (11%)	
*Non-drinker*	2,861 (22%)	357 (33%)	
**Physical.activity**			**0.001**
*non-vigorous activities*	8,151 (70%)	770 (79%)	
*vigorous activities*	2,657 (30%)	182 (21%)	
**Milk.consume**			0.059
*never*	1,687 (15%)	158 (17%)	
*rarely*	1,570 (14%)	132 (13%)	
*sometimes*	3,103 (29%)	238 (23%)	
*often*	4,448 (42%)	424 (47%)	
**Total.calories**	2,001 (1,537, 2,607)	1,746 (1,377, 2,279)	**< 0.001**
**Protein**	78 (59, 103)	67 (52, 89)	**< 0.001**
**Carbohydrate**	239 (182, 310)	225 (173, 294)	**< 0.001**
**Sugars**	101 (69, 146)	104 (69, 145)	0.6
**Fiber**	15 (11, 21)	13 (9, 18)	**< 0.001**
**Fat**	74 (53, 102)	62 (45, 86)	**< 0.001**
**Cholesterol**	242 (155, 373)	197 (136, 316)	**< 0.001**
**Moisture**	2,764 (2,099, 3,657)	2,337 (1,743, 3,104)	**< 0.001**
**Caffeine**	131 (45, 252)	97 (28, 203)	**< 0.001**
**Depression_score**	2.0 (0.0, 4.0)	2.0 (0.0, 6.0)	**< 0.001**
**Depression**	829 (6.6%)	127 (13%)	**< 0.001**
**Major_depression**	302 (2.3%)	51 (5.5%)	**< 0.001**

^1^n (unweighted) (%); Median (IQR)

^2^chi-squared test with Rao & Scott’s second-order correction; Wilcoxon rank-sum test for complex survey samples

### Obtaining Genome-Wide Association Studies (GWAS) data for bi-directional MR

In order to assess the impact of the severity of depression on the analysis results, we selected two sets of depression-related SNPs (depression and major depression) as exposures and chronic constipation-related SNPs as outcomes. The data for depression were obtained from the Medical Research Council-Integrative Epidemiology Unit (MRC-IEU) Consortium, which included 27,568 cases and 457,030 controls. Major depression data were obtained from the Psychiatric Genomics Consortium (PGC), which included 59,851 cases and 113,154 controls. The chronic constipation data also came from the MRC-IEU Consortium, comprising 15,902 cases and 395,721 controls (Supplementary Table S2).

We initially selected SNPs associated with the exposure at a threshold of *P* < 5 × 10^-8, but the resulting IVs were very limited. Having too few IVs may lead to low statistical power and biases such as weak instrument bias. To obtain more accurate and reliable analysis results, we used a threshold of *P* < 5 × 10^-6, which is a standard threshold used in many MR studies [28]. Subsequently, we applied criteria of coefficient LD r^2 < 0.001 and kb = 10,000 to remove linkage disequilibrium effects. To minimize the interference of confounding factors on the analysis results, we queried the selected SNPs strongly associated with the exposure in PhenoScanner and removed those related to risk factors (such as physical activity) for chronic constipation. We harmonized the effect allele for exposure and outcome, removed palindromic SNPs, and excluded SNPs that were incompatible with the outcome. Finally, we calculated the F-statistic for the IVs. To avoid weak instrument bias, we excluded SNPs with F < 10 [29].

### Statistical

In NHANES data analysis, we treated the presence of chronic constipation as a binary categorical variable. We evaluated the correlation between depression scores and the risk of chronic constipation using survey-weighted logistic regression. We also employed RCS to explore potential nonlinear relationships. We designed three models: model 1 without any covariate adjustments, model 2 adjusting for age, sex, race, PIR, BMI, marital status, and education, and model 3, which built upon model 2 by further adjusting for drinking, smoking, physical activity, milk consumption, as well as intake of total calories, protein, carbohydrates, sugar, fiber, fat, cholesterol, moisture, and caffeine. Additionally, we explored interactions between the exposure and covariates on the outcome.

The research methods for the MR analysis adhere to the STROBE-MR checklist [30], as detailed in Supplementary Figure S8. In this analysis, we utilized the IVW, MR-Egger, and weighted median methods. IVW calculates the weight of each IV, effectively reducing the interference of combined effects, and the slope of the corresponding scatter plot reflects the causal relationship trend between the exposure and the outcome. MR-Egger regression provides an effective test for directional pleiotropy, but when pleiotropic instruments are present and the independence assumption is not satisfied, MR-Egger regression cannot provide a consistent estimate of causal effects. The weighted median method is also an MR analysis approach, but it can be biased in the presence of invalid genetic variants. Therefore, we chose IVW as the primary reference method. MR-Egger and the weighted median method were used as supplementary approaches, as they are less efficient but have a wider range of applicability. To enhance the credibility of our results, we conducted rigorous sensitivity analyses. Cochran’s Q was employed for heterogeneity testing. In the presence of heterogeneity, the random-effects IVW was utilized; otherwise, the fixed-effects IVW was applied [31]. MR-Egger and MR-PRESSO methods were used for horizontal pleiotropy detection, and we cross-examined both to obtain the most accurate results [32]. MR-PRESSO also offered outlier and distortion tests to help eliminate SNP outliers [33]. Furthermore, we performed leave-one-out tests to check for the potential influence of individual SNPs on the analysis results. Finally, we conducted reverse MR analysis, with chronic constipation as the exposure and depression as the outcome, to determine the presence of reverse causality. Details can be found in Supplementary Figure S7.

All statistical analyses were carried out using R software version 4.2.2.

## Results

### Participants from the NHANES

Participants were divided into constipated and non-constipated individuals based on their common stool types. Among constipated individuals, there was a higher proportion of females and Non-Hispanic White individuals. The majority of them had a low PIR, high educational attainment, and a higher BMI. Furthermore, there was a higher prevalence of marriage and drinking among constipated individuals, while smoking and vigorous physical activity were less common. Their intake of overall calories, protein, carbohydrates, fiber, fat, cholesterol, moisture, and caffeine was relatively lower, while their sugar intake was higher. Additionally, it is noticeable that constipated individuals had a higher proportion of individuals with depression and major depression (Table 1).

### Correlation of depression and chronic constipation risk in the NHANES analysis

**Fig. 1:**
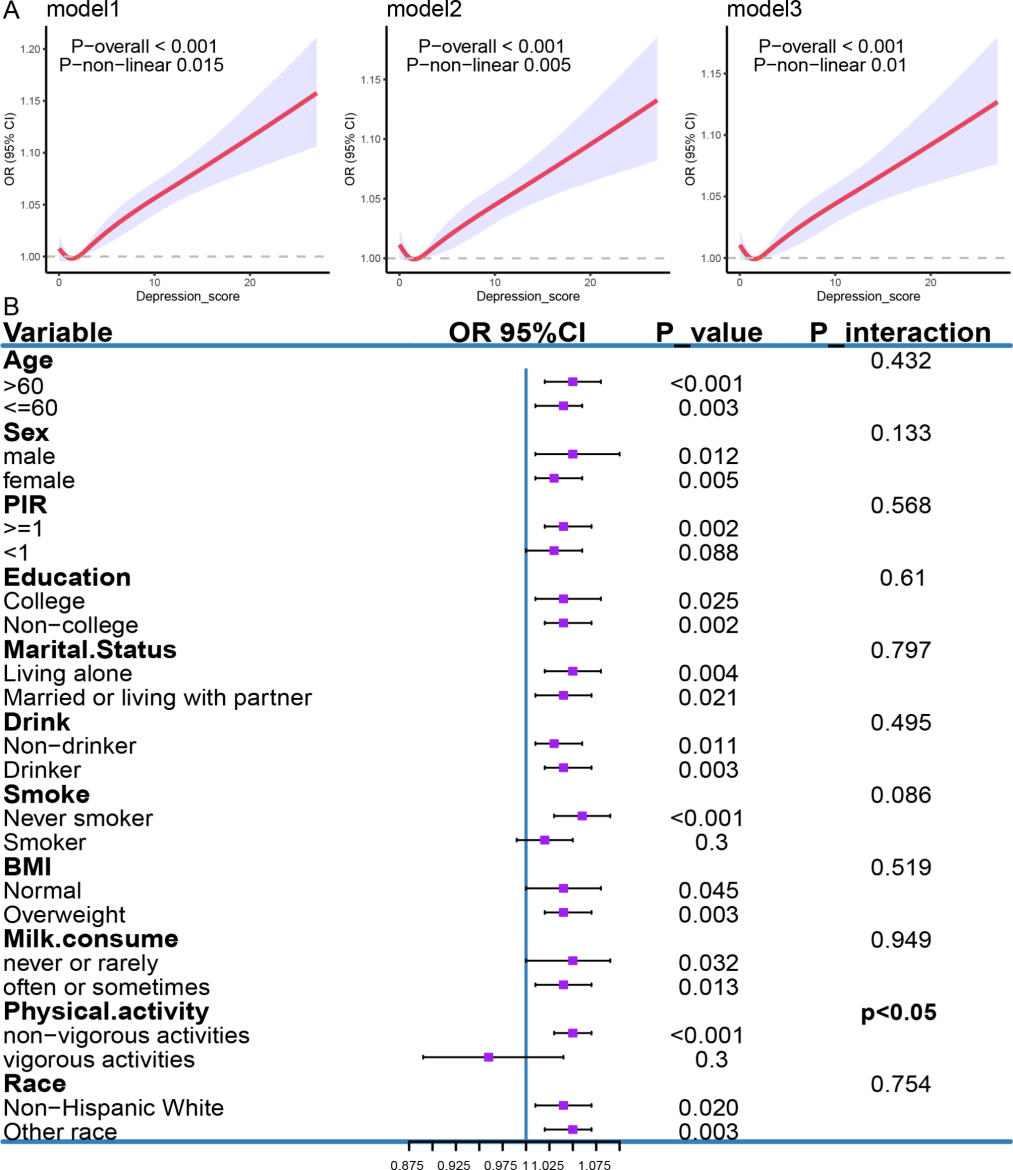
Analysis of RCS and subgroup interactions: (**A**) Nonlinear analysis of the depression scores and the risk of constipation. Model 1: unadjusted. Model 2: adjusted for age, gender, race, PIR, BMI, marital status, and education level. Model 3: adjusted for age, gender, race, PIR, BMI, marital status, education level, drinking, smoking, physical activity, milk consumption, as well as intake of total calories, protein, carbohydrates, sugar, fiber, fat, cholesterol, moisture, and caffeine. (**B**) Interaction analysis between depression scores and age, gender, PIR, education, marital status, alcohol consumption, smoking, BMI, milk consumption, physical activity, and race. *P* < 0.05 is indicated in bold

After adjusting for the included covariates, survey-weighted logistic regression showed that depression scores (OR = 1.04, 95% CI = 1.02–1.07, p = 0.002), depression (OR = 1.66, 95% CI = 1.14–2.42, p = 0.013), and major depression (OR = 1.91, 95% CI = 1.15–3.19, p = 0.017) were all significantly positively associated with the risk of chronic constipation (Table 2). The results of the RCS are similar to those of the logistic regression, showing a significant linear relationship between depression scores and the risk of chronic constipation (P-overall < 0.01) (Fig. 1A). Additionally, the non-linear relationship is also significant (P-nonlinear = 0.01), which may be related to the inclusion of the item ‘poor appetite or overeating’ in the depression score. Both insufficient and excessive food intake could potentially lead to constipation. In the analysis of interactions between depression and covariates, we found that only physical activity interacted with depression scores in relation to the risk of chronic constipation, significantly increasing the likelihood of chronic constipation among individuals lacking vigorous physical activity. However, no significant interactions were observed for the other covariates (Fig. 1B).

**Table 2:** Correlation between depression scores and chronic constipation risk

Characteristic	model1			model2			model3		
	OR^1^	95% CI^1^	*p*-value	OR^1^	95% CI^1^	*p*-value	OR^1^	95% CI^1^	*p*-value
**Depression_score**	1.06	1.04, 1.08	**< 0.001**	1.05	1.02, 1.07	**< 0.001**	1.04	1.02, 1.07	**0.002**
**Depression**	2.10	1.52, 2.90	**< 0.001**	1.74	1.22, 2.47	**0.003**	1.66	1.14, 2.42	**0.013**
**Major_depression**	2.51	1.62, 3.87	**< 0.001**	2.04	1.26, 3.29	**0.005**	1.91	1.15, 3.19	**0.017**

^1^OR = Odds Ratio, CI = Confidence Interval

Model 1: unadjusted. Model 2: adjusted for age, gender, race, PIR, BMI, marital status, and education level. Model 3: adjusted for age, gender, race, PIR, BMI, marital status, education level, drinking, smoking, physical activity, milk consumption, as well as intake of total calories, protein, carbohydrates, sugar, fiber, fat, cholesterol, moisture, and caffeine

### Causal relationships between depression and chronic constipation in MR analysis

**Fig. 2:**
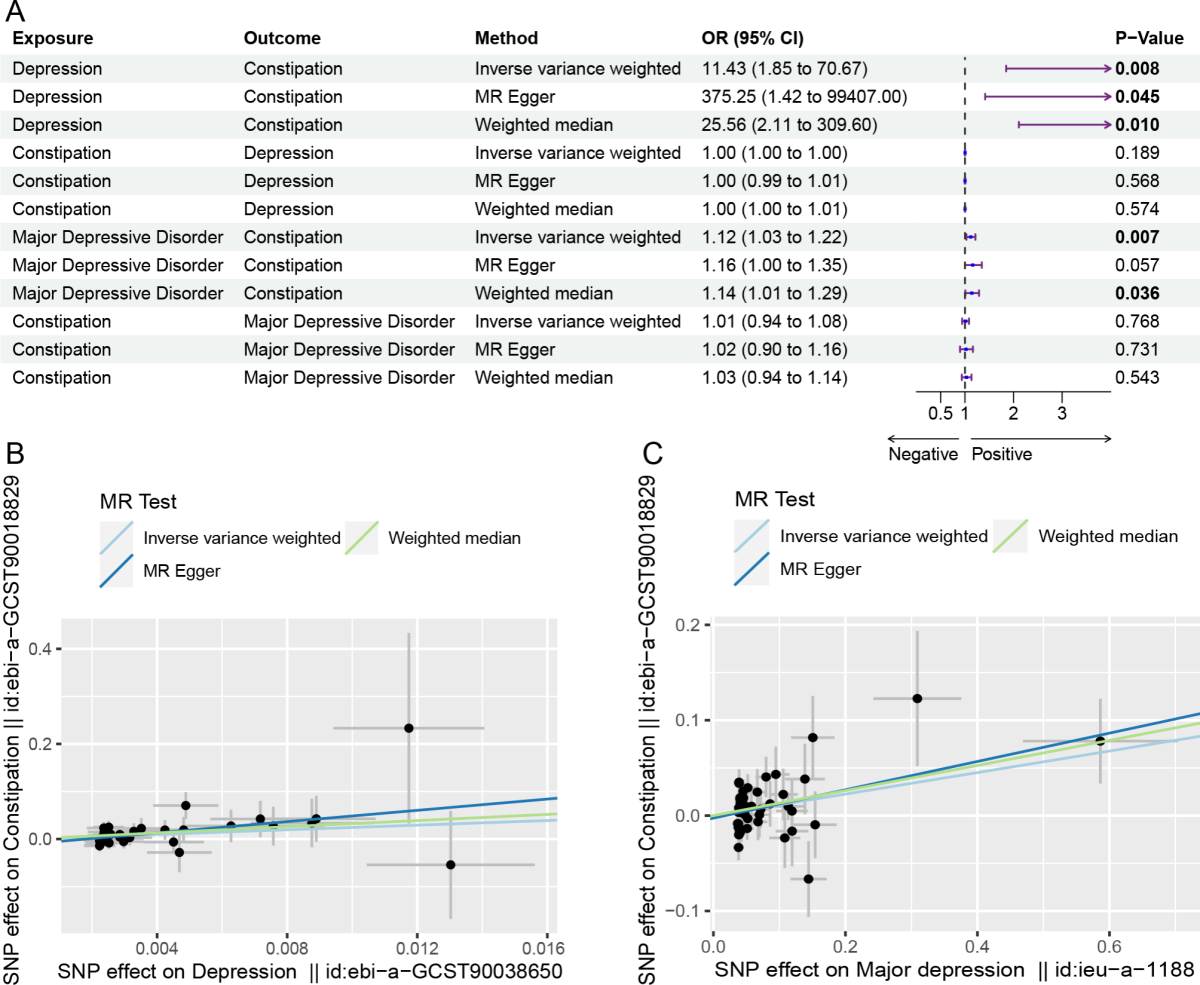
Forest plot and scatter plots for bidirectional two-sample MR analysis: (**A**) Forest plot for bidirectional two-sample MR analysis of depression and major depression with chronic constipation. *P* < 0.05 is indicated in bold. (**B**) Scatter plot for MR analysis between depression and chronic constipation. (**C**) Scatter plot for MR analysis between major depression and chronic constipation

In the previous cross-sectional study, we observed a significant positive correlation between depression scores and the risk of chronic constipation. To validate this correlation, we conducted a causal analysis. The results indicated a causal relationship between depression (OR = 11.43, 95% CI = 1.85–70.67, *p* = 0.008) and major depression (OR = 1.12, 95% CI = 1.03–1.22, *p* = 0.007) and chronic constipation, with both the MR Egger and Weighted median methods yielded similar directions and risk estimate values (Fig. 2A). Figure 2B and C illustrate the trends of the correlation between depression status and chronic constipation. Subsequently, we performed sensitivity tests for heterogeneity and horizontal pleiotropy. For the MR analysis with depression as the exposure, no heterogeneity (*p* = 0.911) or pleiotropy (Pleiotropy-*p* = 0.204, Presso-*p* = 0.939) was detected. It’s worth noting that in the MR analysis with major depression as the exposure, pleiotropy (Pleiotropy-*p* = 0.576, Presso-*p* = 0.063) was not detected, but there was evidence of heterogeneity (*p* = 0.041). Therefore, we employed the random-effects IVW to address this issue (Supplementary Table S3). Furthermore, during the leave-one-out analysis, we did not identify any significantly biased SNPs (Supplementary Figure S2, 4), and the funnel plots exhibited symmetry (Supplementary Figure S6).

### Reverse causality

To explore the existence of a bidirectional causal relationship, we conducted reverse MR analysis with chronic constipation as the exposure and depression as well as major depression as the outcomes. We did not find any significant causal relationships between chronic constipation and either depression (OR = 1.00, 95% CI = 1.00–1.00, *p* = 0.189) or major depression (OR = 1.01, 95% CI = 0.94–1.08, *p* = 0.768) (Fig. 2A). In both reverse analyses, no evidence of heterogeneity or horizontal pleiotropy was detected (Supplementary Table S3), and the leave-one-out analysis and funnel plots did not show any notable anomalies (Supplementary Figure S3, 5, 6). This suggests that there is no reverse causal relationship between depression status and chronic constipation.

## Discussion

In this study, a large-scale observational study within the NHANES cohort (2005–2010) revealed a positive correlation between depression scores and the risk of chronic constipation, even after adjusting for covariates. These findings were corroborated through bidirectional two-sample MR analysis, confirming a significant causal relationship between both depression and major depression with chronic constipation. However, reverse causal analysis did not observe significance, suggesting the absence of a bidirectional causal relationship between depression and chronic constipation.

Due to the comorbidity of depression and chronic constipation, numerous scholars have delved into research on this topic. Ballou et al. found a significant correlation between mild depression and chronic constipation, while there was no significant correlation with moderate to major depression [34]. This contrasts slightly with our observed results. However, their study only utilized data from a single NHANES cycle, which may introduce bias into the results. To obtain more accurate and reliable outcomes, we incorporated data from three cycles of NHANES (2005–2010), revealing a significant association between major depression and chronic constipation. Wang et al. proposed a significant correlation between chronic constipation and depression, suggesting an enhanced screening for depression in chronic constipation patients [35]. We approach this with skepticism as their study was limited to cross-sectional research and did not elucidate whether there is a bidirectional causal relationship. To address this, we conducted bidirectional two-sample MR analysis. The results indicate a significant causal relationship between depression and chronic constipation, with no evidence of a reverse causal relationship. In contrast to their conclusions, this suggests that increasing attention to chronic constipation in patients with depression may be effective clinically, while intensifying depression screening in chronic constipation patients may be ineffective.

To explore the potential causal relationship between the severity of depression and chronic constipation, we included both depression and major depression in the study. Observational studies and MR analysis revealed a positive correlation and causal relationship between both depression and major depression with chronic constipation, but no reverse causal relationship was observed. Interestingly, in the MR analysis, the risk ratio of depression to chronic constipation was significantly higher than that of major depression to chronic constipation (Fig. 2A). This may be due to the limited GWAS data representing depression. Considering both a sufficient sample size and the most up-to-date datasets, obtaining ideal GWAS exposure data is challenging, which could lead to bias in the ORs results. On the other hand, this is likely related to the use of antidepressant medications. Research has found that the benefits of antidepressant medications are more pronounced in patients with major depression compared to those with mild to moderate depression [36]. We speculate that this results in a greater dependence on medication, accompanied by more frequent and higher doses of medication among patients with major depression. Selective serotonin reuptake inhibitors have become a first-line treatment for depression [37]. The neurotransmitter serotonin can regulate the central and enteric nervous systems, influencing gastrointestinal movement and emotions [38, 39]. Israelyan et al. found that serotonin slow-release can restore serotonin in the enteric nervous system, thus treating chronic constipation and gastrointestinal dysfunction [40]. This seems to suggest that taking serotonin-based antidepressants may alleviate chronic constipation in patients with major depression. However, the use of some antidepressants can also increase the risk of constipation. Therefore, we cannot yet draw a conclusion about the relationship between antidepressants and constipation risk. Further mechanistic studies and sufficient cohort studies are needed to explore this issue.

Some studies suggest that female gender and anxiety are significant contributors to chronic constipation [41]. However, our subgroup analysis indicates that only physical activity and depression exhibit an interaction concerning chronic constipation, with the remaining covariates being non-significant. Generally, prolonged sitting and low physical activity are considered risk factors for chronic constipation [42]. Exercise may be an effective preventive measure for chronic constipation [43]. Yet, many studies have not found a significant association between physical activity and chronic constipation, and the relationship between the two requires further validation through additional prospective research [44, 45]. Conversely, the association between physical activity and a reduced risk of depression appears to be more robust. Many scholars propose that physical activity can yield mental health benefits, potentially serving as an effective measure for preventing depression [46, 47]. Therefore, we strongly recommend that individuals, both those with depression and those in good health, maintain regular exercise habits. This not only significantly contributes to mental health but may also help prevent the occurrence of chronic constipation.

Our study used observational research and bidirectional two-sample MR analysis to explore the relationship between depression and chronic constipation. Additionally, we investigated, for the first time, the bidirectional causal relationship between depression, its severity, and chronic constipation. This study has three main strengths. Firstly, the NHANES database’s cohort is sufficiently large, providing reliable analysis results. Secondly, on the basis of cross-sectional studies, we employed two-sample MR analysis for validation and passed rigorous sensitivity checks. MR analysis can effectively avoid confounding factors and reverse causation interference, making our analysis results more robust. Thirdly, we included stratified data on the severity of depression and explored bidirectional causal relationships between different levels of depression and chronic constipation. This contributes to a more accurate and profound understanding of the association between depression and chronic constipation. However, our study also has limitations. Firstly, we used PHQ-9 and BSFS to define depression and chronic constipation. Although these have been proven to be reliable definition standards, bias due to imprecise definitions may still exist. Secondly, despite our comprehensive adjustment for confounding factors, unmeasured or residual confounding effects cannot be entirely ruled out. Thirdly, we aimed to investigate the bidirectional causal relationship between the severity of depression and chronic constipation. However, we did not obtain data on the duration and severity of constipation, so we were unable to explore whether depression exacerbates constipation. Fourth, to avoid the impact of chronic diarrhea on the analysis results, we excluded these participants, which may have limited the representativeness of our study population. Fifth, MR methods can have potential biases, primarily stemming from the possible violations of their fundamental assumptions or difficulties in fully meeting these assumptions in practice. Additionally, the validity of IVW, MR-Egger, and weighted median methods can vary under different conditions or assumptions, which may also introduce potential biases. Lastly, our study only involved populations from the United States and Europe. Further research involving more diverse racial and ethnic groups is needed, but our findings can provide direction for future studies.

## Conclusions

Through cross-sectional research and MR analysis, we found a positive correlation between depression, including major depression, and chronic constipation. This suggests that in clinical practice, it may be effective to enhance constipation screening in patients with depression, consider prophylactic use of medications, and advise patients to consume easily digestible foods to prevent the progression and worsening of constipation.

## Electronic supplementary material

Below is the link to the electronic supplementary material.

Supplementary Material 1: The supplementary materials include data sources, flowcharts, leave-one-out tests, funnel plots, and STROBE-MR checklist

## Data Availability

All data used in this study can be accessed at https://wwwn.cdc.gov/nchs/nhanes/Default.aspx and https://gwas.mrcieu.ac.uk/.

## Abbreviations

NHANES: National Health and Nutrition Examination Survey
MR: Mendelian randomization
IVW: Inverse Variance Weighting
RCS: Restricted Cubic Splines
SNPs: Single nucleotide polymorphisms
IVs: Instrumental variables
PIR: Poverty income ratio
BMI: Body Mass Index
PHQ: Patient Health Questionnaire
BSFS: Bristol Stool Form Scale
GWAS: Genome-wide association studies
MRC-IEU: Medical Research Council-Integrative Epidemiology Unit
PGC: Psychiatric Genomics Consortium
