# Supplementary material for: Can depression lead to chronic constipation, or does chronic constipation worsen depression? NHANES 2005–2010 and bidirectional mendelian randomization analyses
Source: BMC Gastroenterol. 2024 Oct 10;24:361. doi: 10.1186/s12876-024-03454-x (PMC11468412; doi:10.1186/s12876-024-03454-x)
Supplement: Supplementary file 1 — Supplementary Material 1: The supplementary materials include data sources, flowcharts, leave-one-out tests, funnel plots, and STROBE-MR checklist [file 12876_2024_3454_MOESM1_ESM.docx]

**Supplementary Table S1** Correlation between depression scores and chronic diarrhea risk.

|  | model1 | | | model2 | | | model3 | | |
| --- | --- | --- | --- | --- | --- | --- | --- | --- | --- |
| **Characteristic** | **OR**^1^ | **95% CI**^1^ | **p-value** | **OR**^1^ | **95% CI**^1^ | **p-value** | **OR**^1^ | **95% CI**^1^ | **p-value** |
| **Depression_score** | 1.08 | 1.06, 1.11 | **<0.001** | 1.07 | 1.05, 1.10 | **<0.001** | 1.07 | 1.04, 1.10 | **<0.001** |
| **Depression** | 2.78 | 1.98, 3.90 | **<0.001** | 2.40 | 1.67, 3.47 | **<0.001** | 2.28 | 1.51, 3.45 | **0.001** |
| **Severe_depression** | 3.09 | 2.06, 4.63 | **<0.001** | 2.53 | 1.64, 3.92 | **<0.001** | 2.36 | 1.49, 3.75 | **0.002** |
| ^1^OR = Odds Ratio, CI = Confidence Interval | | | | | | | | | |

**Supplementary Table S2** Details of GWAS data for depression, major depression, and constipation.

| **Phenotype** | **Year** | **Participants** | **Ncase** | **Ncontrol** | **Source** |
| --- | --- | --- | --- | --- | --- |
| Depression | 2021 | 484,598 | 27,568 | 457,030 | [https://gwas.mrcieu.ac.uk/datasets/ebi-a-GCST90038650/](https://gwas.mrcieu.ac.uk/datasets/ebi-a-GCST90038650/) |
| Constipation | 2021 | 411,623 | 15,902 | 395,721 | [https://gwas.mrcieu.ac.uk/datasets/ebi-a-GCST90018829/](https://gwas.mrcieu.ac.uk/datasets/ebi-a-GCST90018829/) |
| Major Depressive Disorder | 2018 | 173,005 | 59,851 | 113,154 | [https://gwas.mrcieu.ac.uk/datasets/ieu-a-1188/](https://gwas.mrcieu.ac.uk/datasets/ieu-a-1188/) |

**Supplementary Table S3** Sensitivity testing for bidirectional two-sample MR analysis of depression, major depression, and constipation.

| **Causal analysis** | **Heterogeneity-pval** | **Pleiotropy-pval** | **Presso-pval** |
| --- | --- | --- | --- |
| Depression-Constipation | 0.911 | 0.204 | 0.939 |
| Constipation-Depression | 0.349 | 0.83 | 0.391 |
| Major Depressive Disorder-Constipation | 0.041 | 0.576 | 0.063 |
| Constipation-Major Depressive Disorder | 0.79 | 0.824 | 0.793 |


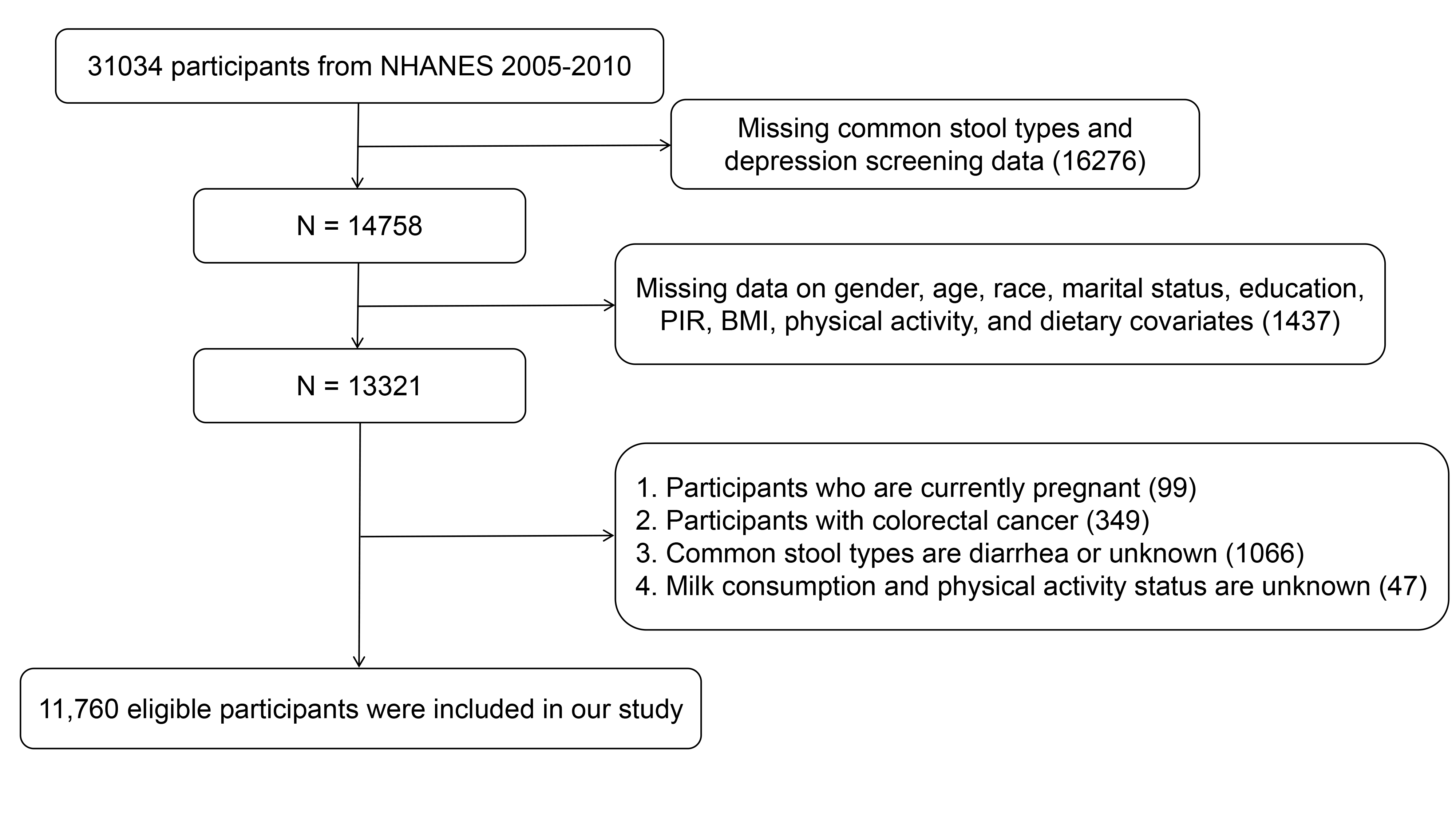


**Supplementary Figure S1** Flowchart of the NHANES analysis.


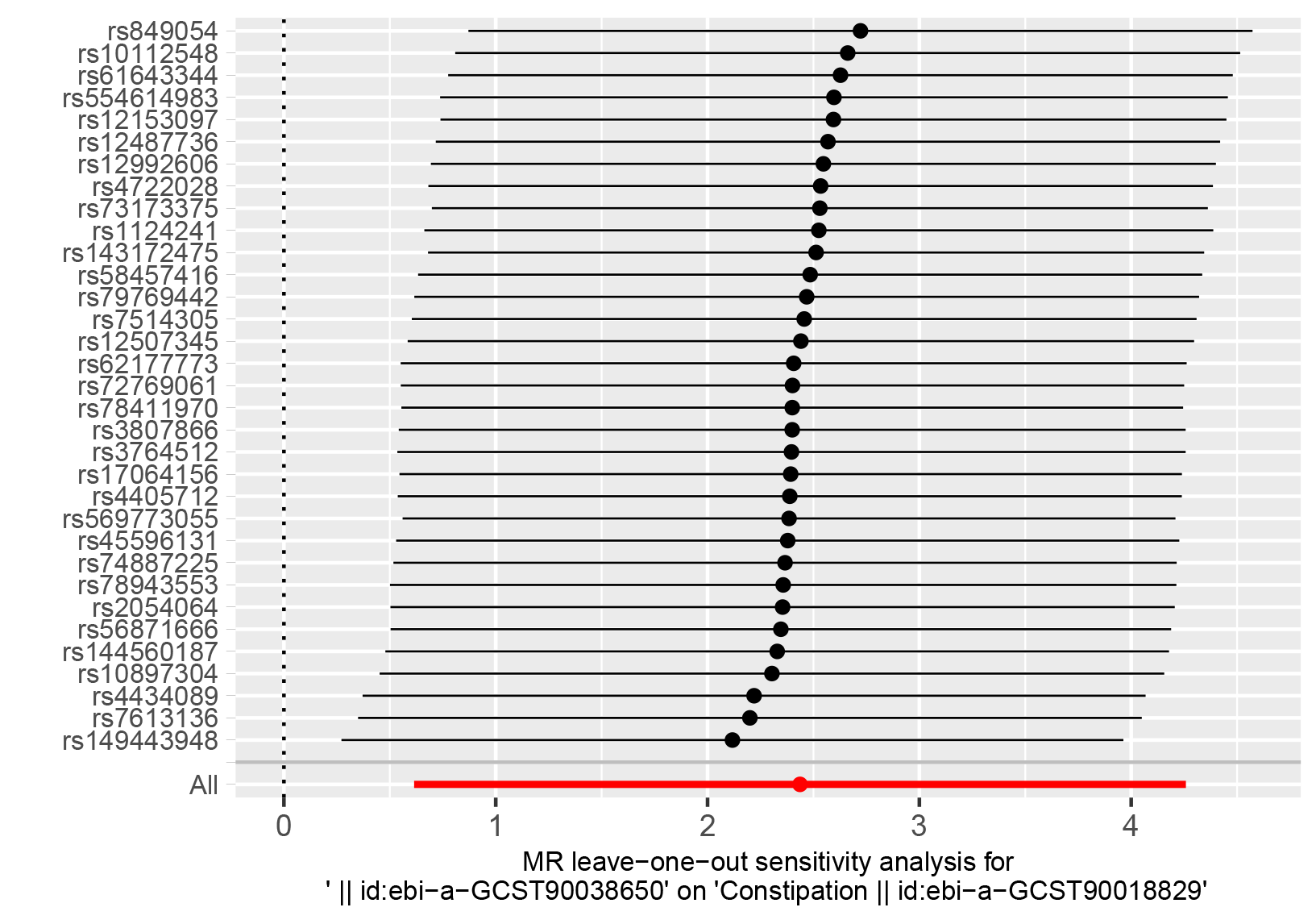


**Supplementary Figure S2** Leave-one-out method test for MR analysis of depression and constipation.


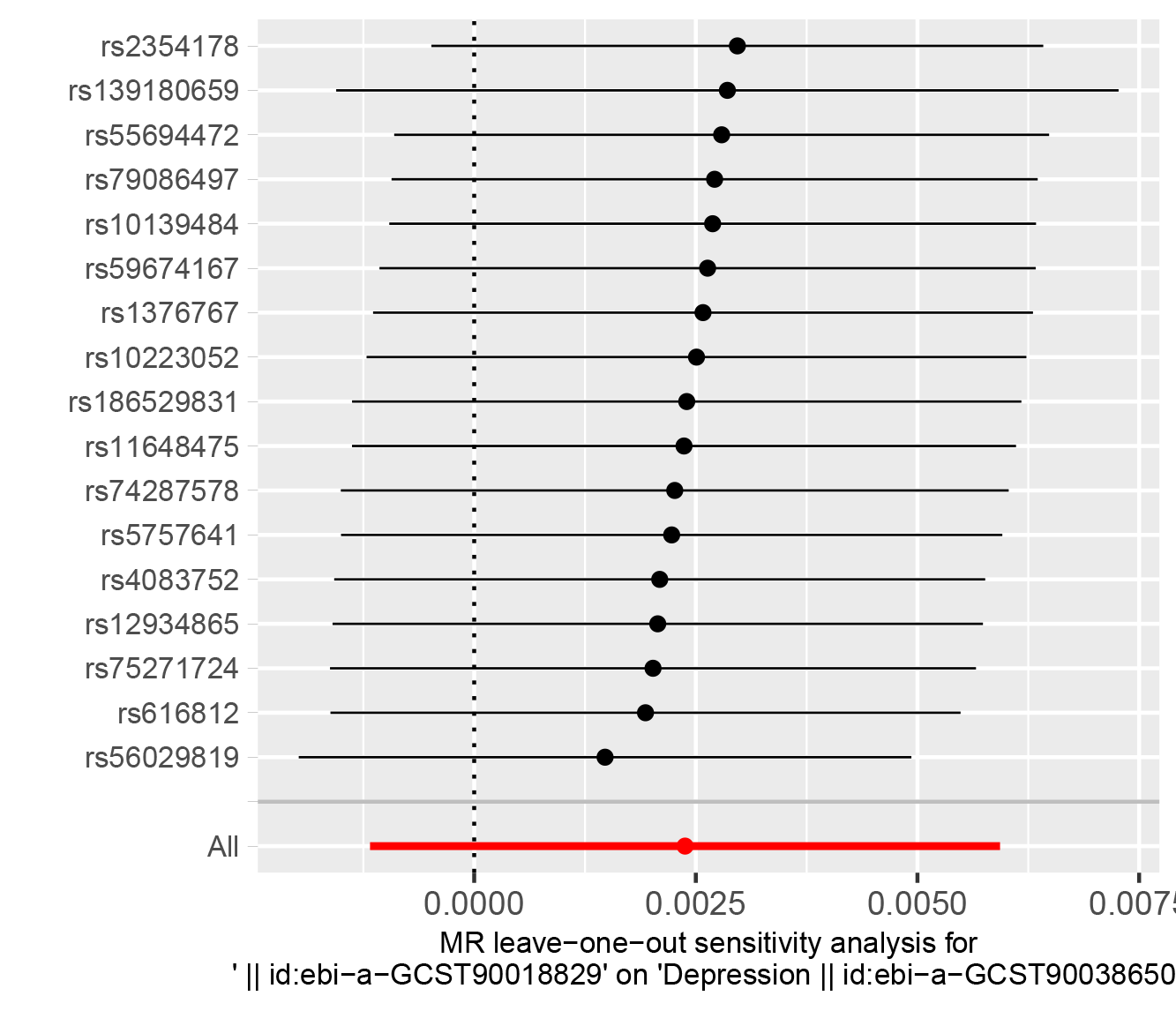


**Supplementary Figure S3** Leave-one-out method test for the reverse MR analysis of depression and constipation.


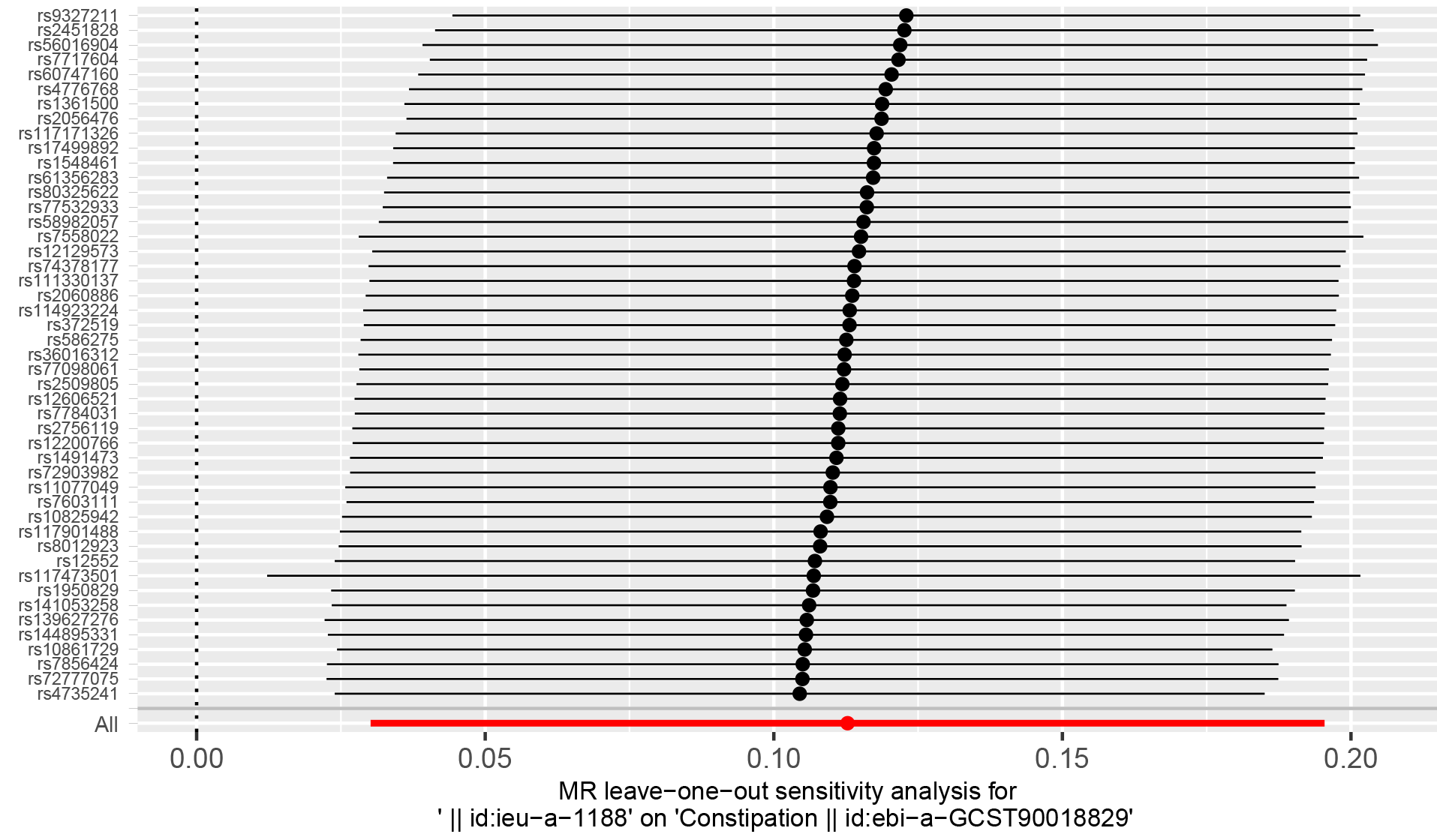


**Supplementary Figure S4** Leave-one-out method test for MR analysis of major depression and constipation.


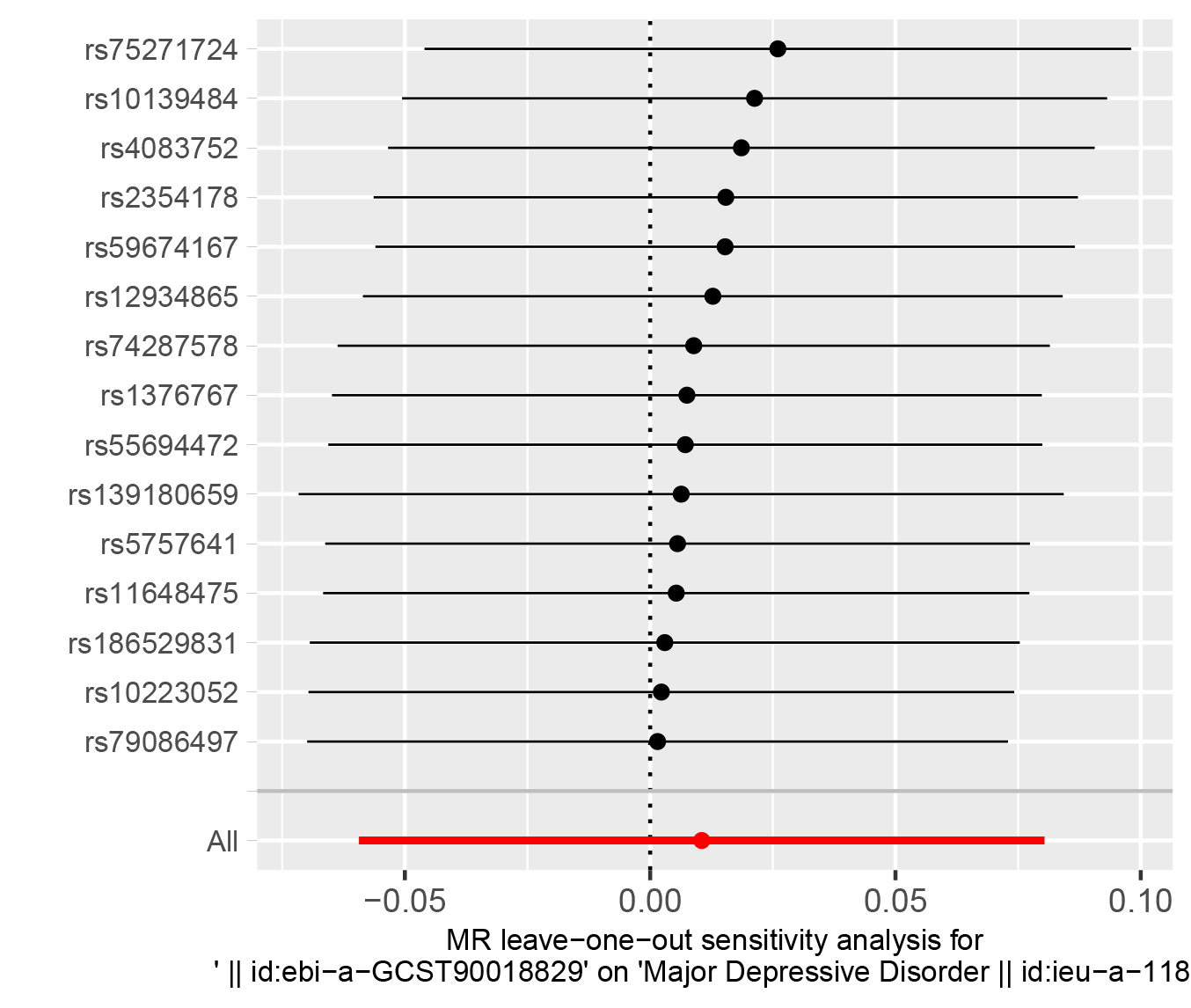


**Supplementary Figure S5** Leave-one-out method test for the reverse MR analysis of major depression and constipation.


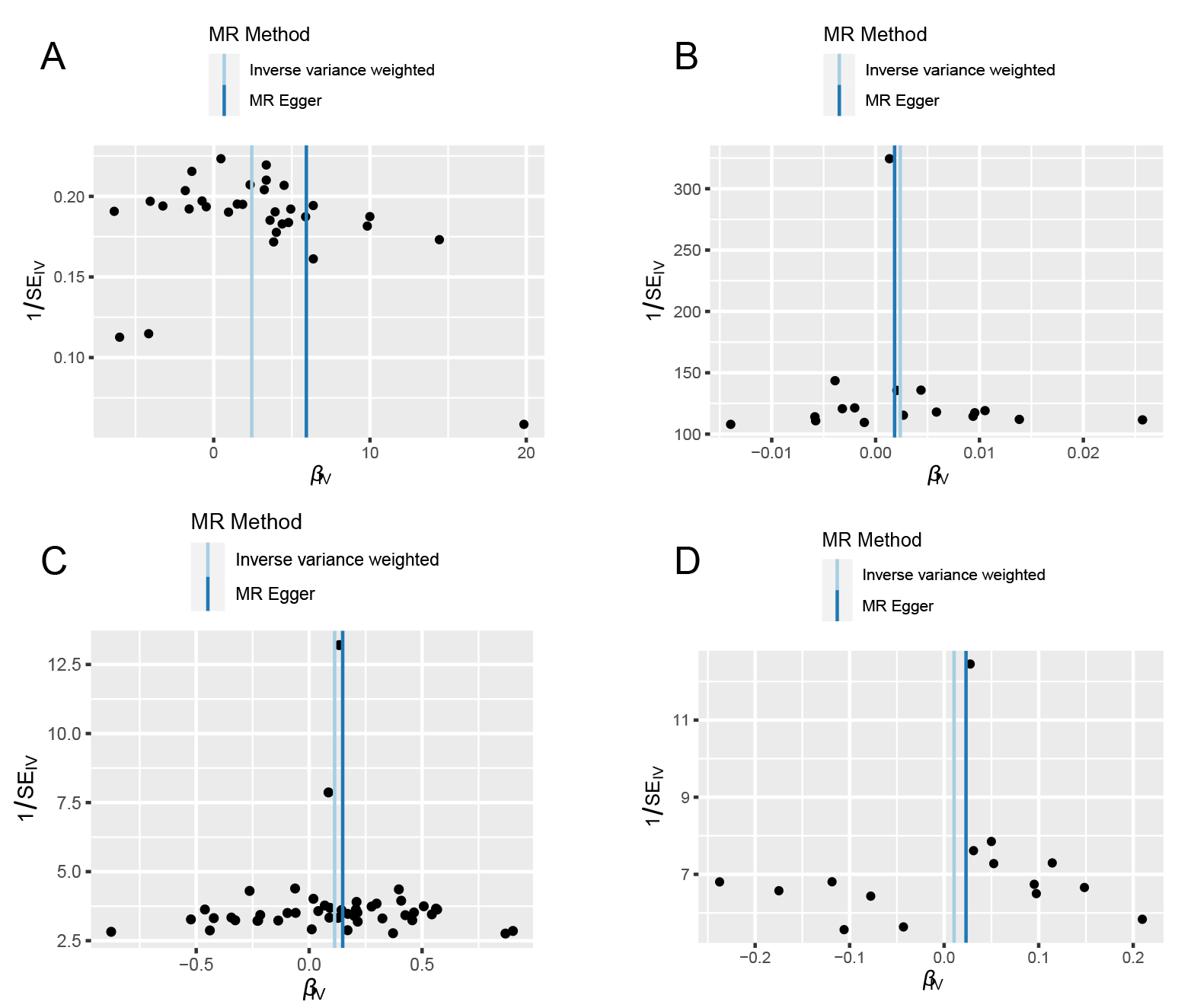


**Supplementary Figure S6** Funnel plots for MR analysis: (A) Depression and constipation. (B) Reverse analysis of depression and constipation. (C) Major depression and constipation. (D) Reverse analysis of major depression and constipation.


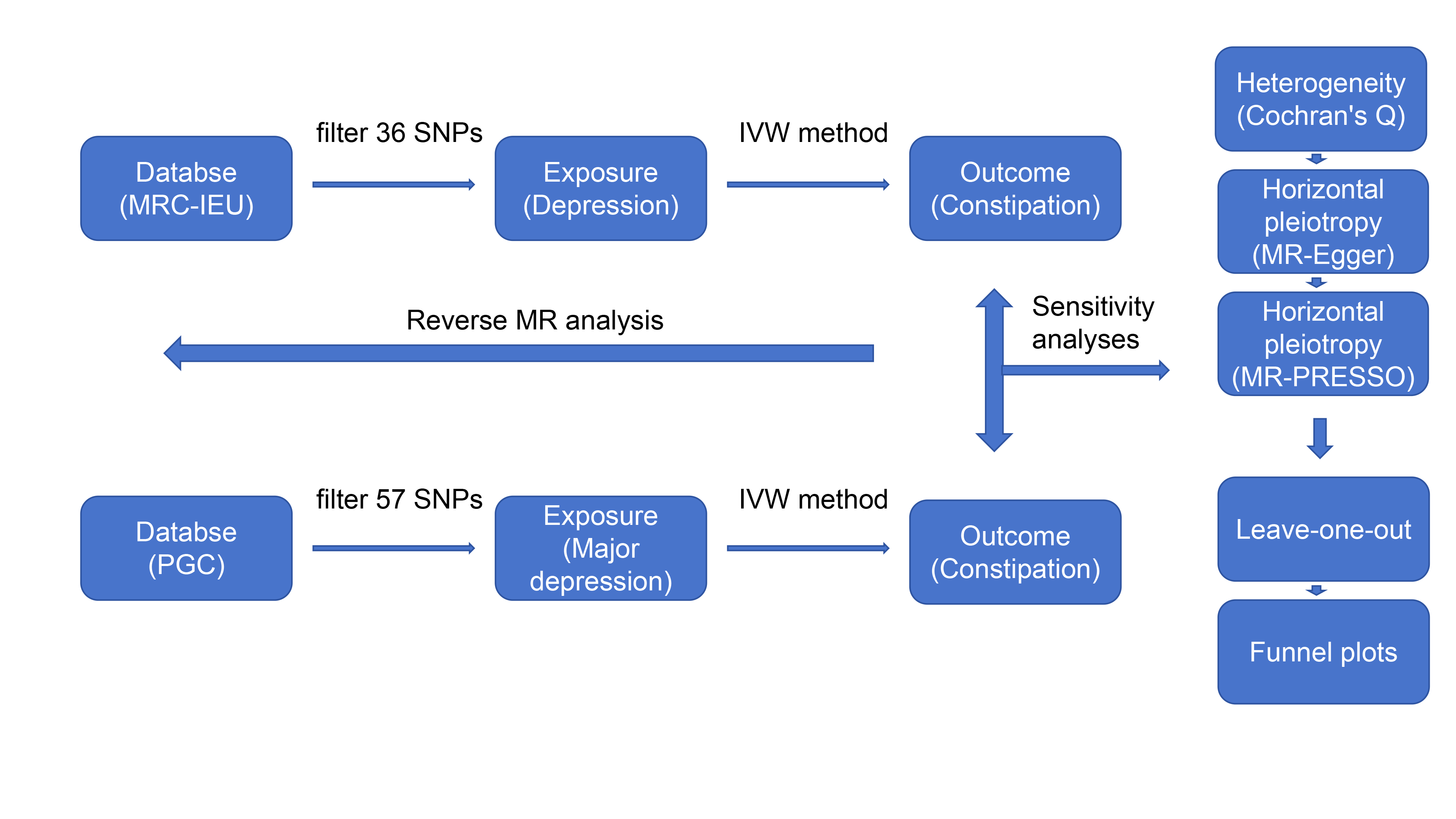


**Supplementary Figure S7** Detailed flow chart of the MR analysis.

**
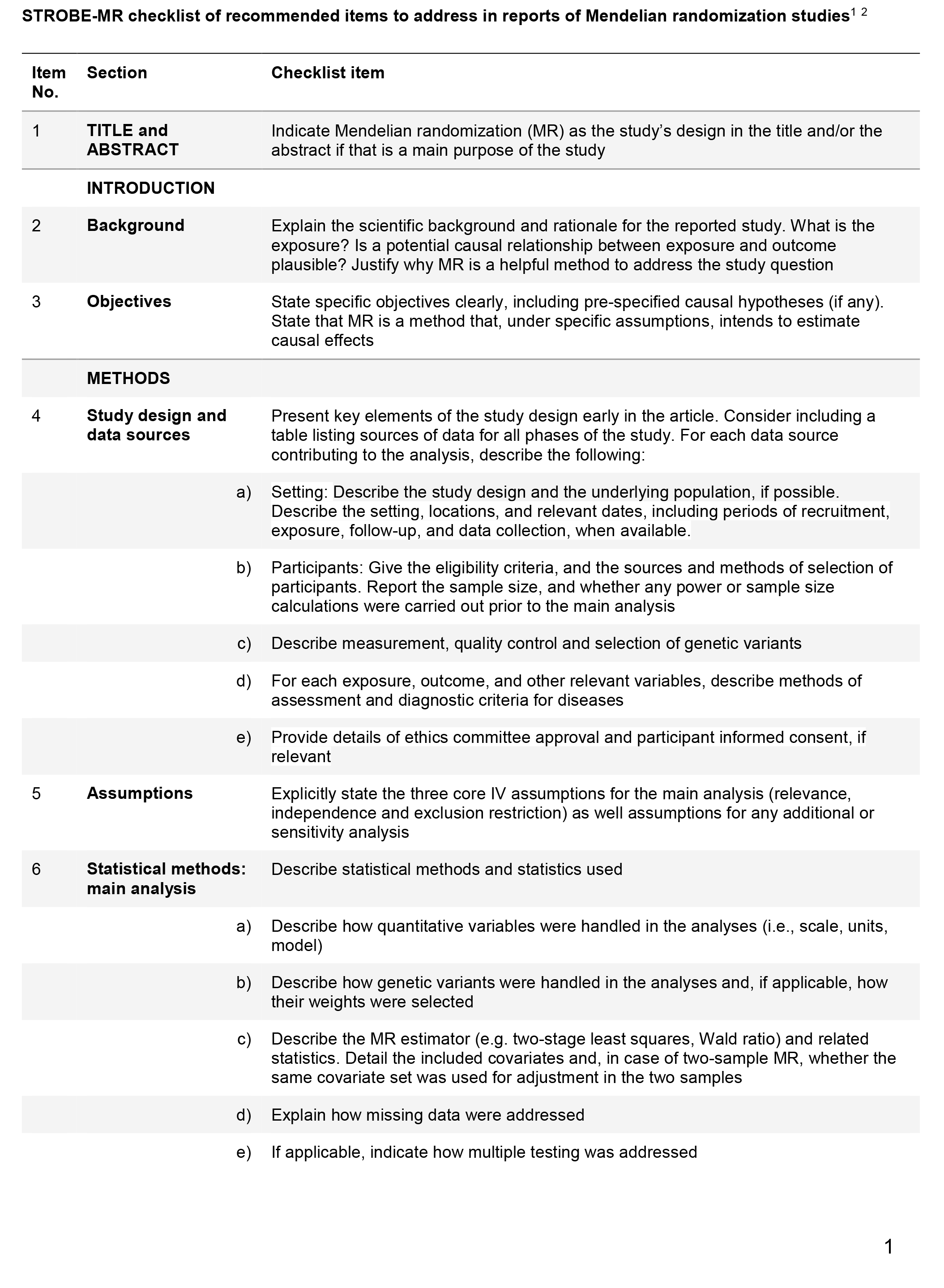

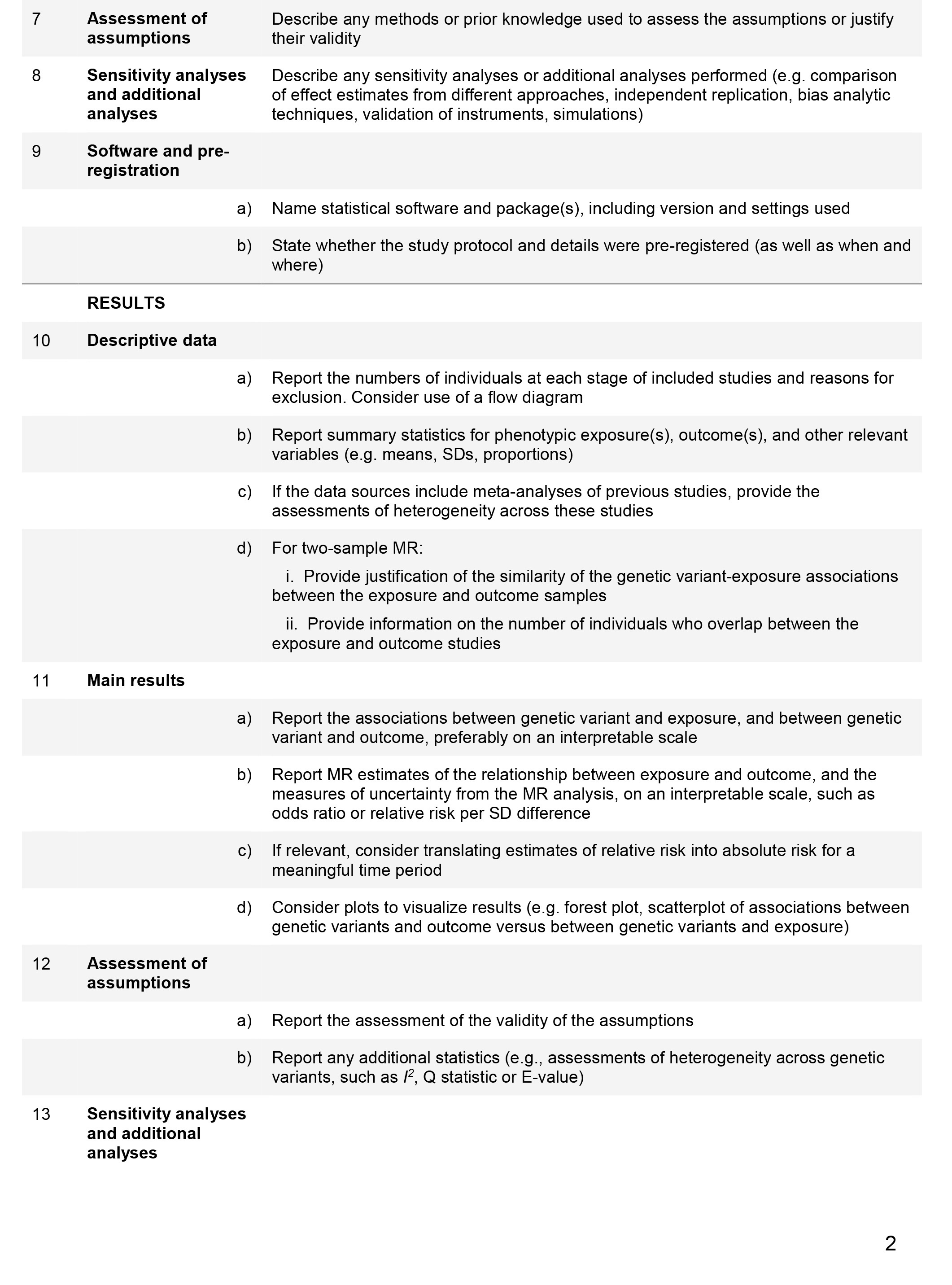

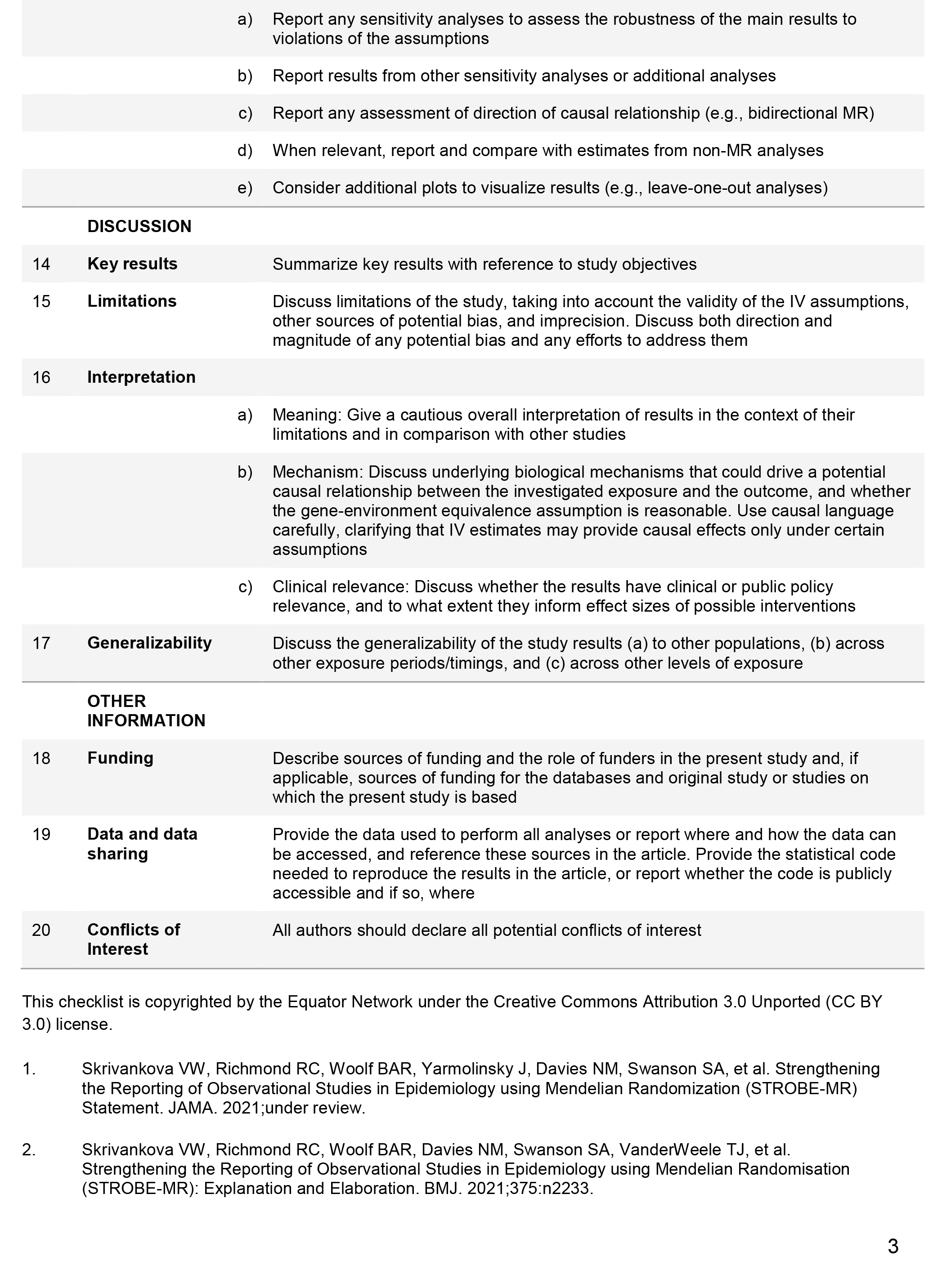
**

**Supplementary Figure S8** STROBE-MR checklist of recommended items to address in reports of Mendelian randomization studies.
